# Leishmanicidal Activity of Withanolides from *Aureliana Fasciculata* var. *Fasciculata*

**DOI:** 10.3390/molecules23123160

**Published:** 2018-11-30

**Authors:** Simone Cristina de M. Lima, Juliana da Silva Pacheco, André M. Marques, Eduardo Raul Pereira Veltri, Rita de Cássia Almeida-Lafetá, Maria Raquel Figueiredo, Maria Auxiliadora Coelho Kaplan, Eduardo Caio Torres-Santos

**Affiliations:** 1 Instituto de Pesquisas de Produtos Naturais (IPPN), Universidade Federal do Rio de Janeiro, Av. Carlos Chagas Filho-Cidade Universitária, 21941-902 Rio de Janeiro, Brazil; smnioe@gmail.com (S.C.d.M.L.); makaplan@nppn.ufrj.br (M.A.C.K.); 2 Laboratório de Bioquímica de Tripanosomatídeos, Instituto Oswaldo Cruz, Fundação Oswaldo Cruz (FIOCRUZ), 210360-040 Rio de Janeiro, Brazil; juspacheco@hotmail.com (J.d.S.P.); erpv1994@gmail.com (E.R.P.V.); 3 Departamento de Produtos Naturais, Farmanguinhos, Fundação Oswaldo Cruz (FIOCRUZ), 21041-250 Rio de Janeiro, Brazil; andrefarmaciarj@yahoo.com.br (A.M.M.); mraquelf6@yahoo.com.br (M.R.F.); 4 Faculdade de Educação Tecnológica do Rio de Janeiro (FAETERJ-Rio), 21351-290 Rio de Janeiro, Brazil; ritalafeta@yahoo.com.br

**Keywords:** leishmania, solanaceae, withanolides, aurelianolides

## Abstract

Leishmaniasis is the generic denomination to the neglected diseases caused by more than 20 species of protozoa belonging to the genus *Leishmania*. The toxic and parenteral-delivered pentavalent antimonials remain to be the first-line treatment. However, all the current used drugs have restrictions. The species *Aureliana fasciculata* (Vell.) Sendtner var. *fasciculata* is a native Brazilian species parsimoniously studied on a chemical point of view. In this study, the antileishmanial activity of *A. fasciculata* was evaluated. Among the evaluated samples of the leaves, the dichloromethane partition (AFfDi) showed the more pronounced activity, with IC_50_ 1.85 µg/ml against promastigotes of *L. amazonensis*. From AFfDi, two active withanolides were isolated, the Aurelianolides A and B, with IC_50_ 7.61 μM and 7.94 μM, respectively. The withanolides also proved to be active against the clinically important form, the intracellular amastigote, with IC_50_ 2.25 μM and 6.43 μM for Aurelianolides A and B, respectively. Furthermore, withanolides showed results for in silico parameters of absorption, distribution, metabolism, excretion, and toxicity (ADMET) similar to miltefosine, the reference drug, and were predicted as good oral drugs, with the advantage of not being hepatotoxic. These results suggest that these compounds can be useful as scaffolds for planning drug design.

## 1. Introduction

Leishmaniasis is the generic denomination to the diseases caused by more than 20 species of protozoa belonging to the genus *Leishmania*. Cutaneous leishmaniasis (CL) is the most prevalent form, caused mainly by *L. major* and *L. tropica* in Old World and by *L. braziliensis*, *L. guyanensis*, and *L. amazonensis* in New World, with an estimated 0.6 million to 1 million new cases occurring worldwide annually [1]. The toxic and parenteral-delivered pentavalent antimonials (N-methylglucamine antimoniate and sodium stibogluconate) remain to be the first-line treatment for CL in most countries. In addition, amphotericin B (conventional and liposomal) is used as an alternative in cases of unresponsiveness. The Food and Drug Administration (FDA) authorizes the use of miltefosine, the only oral drug available, for all clinical manifestations of leishmaniasis in the United States, including CL, but its efficacy in some endemic countries in South America is variable [2]. Other drugs introduced for CL treatment include pentamidine and paromomycin. In milder cases, the use of antimony in combination with cryotherapy is recommended [3]. However, all these drugs may lead to serious side effects, high toxicity or induction of parasite resistance [4,5,6].

The Solanaceae Family is considered one of the largest families among the eucotiledonous angiosperms, gathering around 150 genera and 3000 species concentrated in the neotropical region [7]. The species of this family are of great economic importance, being used in food such as potato (Solanum tuberosum), tomato (*Solanum lycopersicum*), eggplant (*Solanum melongena*), and pepper (*Capsicum annum*) [8]. Several of these species have been investigated with great interest by the pharmaceutical industry due to their bioactive metabolites, such as alkaloids and steroids that occur in many genera [9].

The genus *Aureliana* is a small endemic genus in the Solanaceae family, widely distributed in South and Southeastern of Brazil, usually found in mountain forests area, semi-deciduous mesophyllous forests, and reef areas [10]. The species *Aureliana fasciculata* (Vell.) Sendtner var. *fasciculata* is a native Brazilian species rarely studied on a chemical point of view, which is found in Atlantic Forest [11].

Phytochemical studies showed that the steroid derivatives are the major compound metabolites present in *A. fasciculata* leaves [11]. The withasteroids comprises a group of steroidal substances characterized by a moiety ergostane with 28 carbon atoms, where C-22 and C-26 are oxidized to form six-membered lactone [12]. The most abundant type is usually designated as withanolides and these compounds possess an α,β-unsaturated δ-lactone ring in the side chain of the molecule [13]. These steroid derivatives are frequently polyoxygenated, and biogenetic transformations can produce highly modified structures, both in the steroid nucleus and in the side chain [14].

Since the 1960s, about 750 withanolides have been isolated. These substances are found in many genera of the family Solanaceae, such as *Acnistus*, *Datura*, *Deprea*, *Discopodium*, *Dunalia*, *Iochroma, Jaborosa, Lycium, Nicandra, Physalis, Solanum, Trechonaetes, Tubocapsicum, Vassobia, Withania*, and *Witheringia* [15]. However, the occurrence of withanolides is not completely restricted to Solanaceous plants and reports of their isolation from marine organisms, and from members of the Taccaceae, Fabaceae (Leguminosae) [16], and Dioscoreaceae [17], Myrtaceae and Lamiaceae [18] families suggest that they are much more widely distributed.

In this work, the antileishmanial property of *Aureliana fasciculata* Vell. Sendtner var. *fasciculata* was first demonstrated and the purification guided by the biological activity pointed two withanolides as the active constituents.

## 2. Results and Discussion

**Figure 1:**
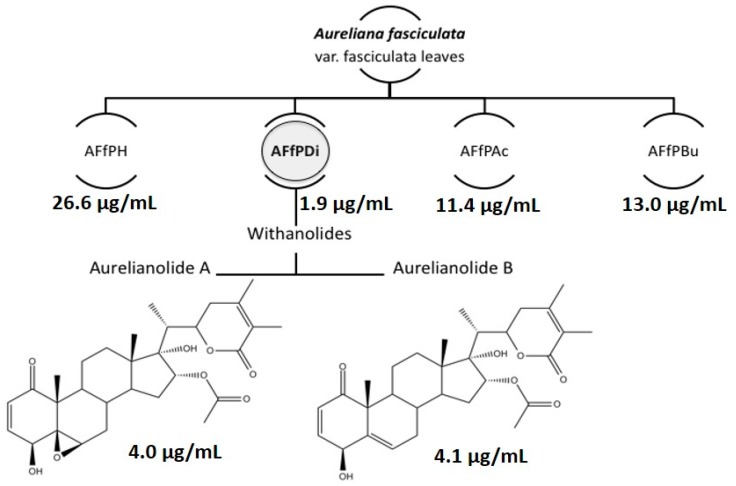
Antileishmanial-guided extraction of leaves of *Aureliana fasciculata*. AFfPH, hexane partition; AFfPDi, dichloromethane partition; AFfPAc, ethyl acetate partition; AFfPBu, butanol partition. The numbers refer to antipromastigote ICs_50_.

Following an approach of antileishmanial-guided extraction, the methanolic extract of leaves of *A. fasciculata* was submitted to partition using solvents with crescent polarities (Figure 1). The resulting fractions were evaluated for antipromastigote activity. All fractions showed some degree of promastigote inhibition, but only the dichloromethane fraction (AFfPDi) had IC_50_ below than 10 µM (1.85 µM), the threshold considered in this study. Thus, this fraction was successively chromatographed, originating two purified withanolides, Aurelianolide A and Aurelianolide B.

These compounds were first isolated from the same species and described by Almeida-Lafetá in 2010 [11] (Figure 1).

The Aurelianolide A (MW: 528,64) was obtained as a white amorphous solid and its molecular formula was deduced as C_32_H_38_O_8_. The mass spectra of HRMS showed an ion with m/z 551.2682 resulting from the formation of adducts of the Aurelianolide A with sodium ions. The Aurelianolide B (MW: 512,64) was obtained as white crystals having its molecular formula deduced as C_32_H_39_O_7_. The mass spectra of HRMS showed an ion with m/z 535.2682 resulting from the formation of adducts of one substance with sodium ions. The NMR data were compared to the literature data [11]. Up to this moment, no biological studies were performed with these steroid-derivatives metabolites.

The purified compounds conserved the antipromastigote activity, although being slightly less active separately, with IC_50_ of 4.0 µg/ml (7.6 µM) and 4.1 µg/ml (7.9 µM), for Aurelianolides A and B, respectively (Figure 1, Table 1).

Following these results, Aurelianolide A and Aurelianolide B were evaluated for antimastigote activity, the clinically relevant form. Both compounds were active but, differently of the antipromastigote action, they had distinct potencies. Aurelianolide A was more potent, with an IC_50_ 1.2 μg/ml (2.3 μM), while Aurelianolide B showed an IC_50_ 3.3 μg/ml (6.43 μM).

Withanolides have already shown to possess various biological activities such as anti-inflammatory [19,20], antitumoral [20], trypanosomicidal [21], antileishmanial [22], immunomodulatory [23], and antibacterial [24]. Such substances also exhibit insecticidal activity and phytotoxicity [25]. Some withanolides with highlighted leishmanicidal activity were isolated from *Withania coagulans* [26], *Physalis minima* [27], and *Dunalia brachyachantha* [28].

To evaluate the selectivity, the cytotoxic profile against J774 cells was evaluated with resazurin. Both Aurelianolides A and B showed the same cytotoxic activity to J774 macrophages, with CC_50_ 6.7 µg/ml (12.7 µM and 13.1 µM, respectively) (Table 1). Note that, in this case, the presence of the epoxide did not influence in the activity, as well as in promastigotes. The selectivity index (SI) was also calculated, revealing an SI for Aurelianolide A of 5.6 and for Aurelianolide B of 2.0. The calculated SI for Aurelanolide A was higher than that found for the reference drug, pentamidine (4.5, Table 1).

**Figure 2:**
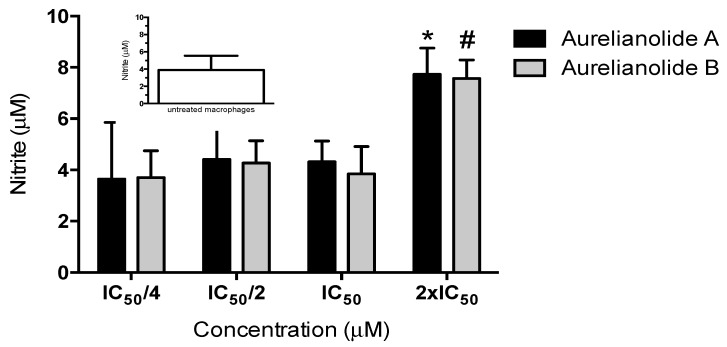
Effect of Aurelianolides on Nitric Oxide production by macrophages infected with *L. amazonensis.* Concentrations are related with a quarter, half, twice and the IC_50_ values of antipromastigote activity.

The higher activity of withanolides in intracellular amastigotes, mainly the Aurelianolide A, is suggestive that the host cell could be playing a role in clearing the parasites. Nitric oxide (NO) is an important tool for killing intracellular parasites. The outcome of treatment with withanolides was also examined by measuring the NO concentration on the culture supernatant. Figure 2 shows that the NO level increased significantly when infected macrophages were treated with twice the IC_50_ values of antipromastigote activity for both withanolides. The NO level was low among the infected macrophages treated with a quarter the IC_50_ value of Aurelianolide A, but there were no significant differences.

NO, produced by the nitric oxide synthase (iNOS) enzyme, is a product of macrophages activated by cytokines and is one of the most important molecules responsible for the killing of *Leishmania* parasites [29,30]. It was observed that IFN-γ has been shown to synergize with TNF in murine systems, leading NO production by iNOS, resulting in eradication of intracellular parasites. In the infected host organisms, functions of NO described to date include antiviral, antimicrobial, immunostimulatory (proinflammatory), immunosuppressive (anti-inflammatory), cytotoxic (tissue-damaging), and cytoprotective (tissue-preserving) effects. The antimicrobial activity of NO was originally thought to result from mutation of DNA, inhibition of DNA repair and synthesis, inhibition of protein synthesis and alteration of proteins by S-nitrosylation, ADP-ribosylation, or tyrosine nitration [31]. Previous findings show a higher NO expression in monocytes from human CL patients comparing to expression in monocytes from healthy patients. It was observed a huge correlation between NO production and lesion size of CL patients. Further, NO alone is not sufficient to control infection and may contribute to the tissue damage observed in human CL [32].

Three other withanolides isolated from leaves of Solanaceae family were found to be responsible for inhibiting NO production by activated macrophages [33]. In this work, NO production in infected macrophages treated with a quarter, half, and the IC_50_ value of withanolides did not show significant differences comparing the NO levels of non-treated infected macrophages. The NO production by infected cytokines-activated macrophages and its consequences in killing parasites should be further investigated.

The theoretical analysis of the physicochemical parameters, Lipinski’s rule of five (Ro5) and ADMET (absorption, distribution, metabolism, excretion, and toxicity) properties of withanolides and miltefosine were performed using the Predicting Small-Molecule Pharmacokinetic and Toxicity Properties (PkCSm tool). In short, the rule of five predicts absorption or drug permeability parameters which are values multiple of 5 or even 5 [34]. The comparison with miltefosine was made since is the only oral drug available for leishmaniasis treatment [35]. In the first analysis, we observed that both withanolides violated for a few tens only one parameter; the molecular weight that should be below 500. Also, a little violation was observed for miltefosine in the logP parameter with a value above 5, while Aurelianolide A and Aurelianolide B followed the rule proposal. Taken together with the water solubility values found, these results suggest a profile of water-soluble drugs for withanolides (Table 2). Mckerrow and Lipinski (2017) [35] highlighted that they never intend the “rule of 5” as a mainstay fixed rule for a new drug, but a parameter to be carefully evaluated. Miltefosine represented a breakthrough, as the first orally active compound for Leishmaniasis in clinics and since then screening efforts continue the search for an oral improved drug when it comes parasitic diseases drug discovery in general [36,37,38].

The in silico ADMET analysis (Table 3) showed a good probability of permeability on Caco2 cells, with values above of the adopted threshold of 0.9 for Aurelianolide A and B. High human intestinal absorption probability was observed for Aurelianolide A (91%) and Aurelianolide B (90.53%), near to values found for miltefosine (94.987%). The withanolides showed low distribution volume (VDss less than 0.56 l/kg), suggesting low output from blood to the tissues. Furthermore, both withanolides are unlikely to penetrate the central nervous system (CNS) (logBBB < 0 and logPS < −2), which helps to reduce side effects and toxicity. The prediction suggests withanolides should be metabolized by cytochrome P3A4 (CYP3A4), and there is no indication of inhibition of the main cytochrome P450 (CYP P450) oxidases.

Toxicity predictions (Table 4) pointed out that Aurelianolides and miltefosine are not likely to be mutagenic or cause skin sensitization. Predictions suggest that equally to miltefosine, Aurelianolides are not expected to inhibit human ether-à-go-go related genes (hERGI), but probably inhibit hERGII. These predictions indicate the need for evaluation of cardiac markers in the biochemical analysis when we proceed to tests in animal model. Aurelianolides were not predicted to be hepatotoxic unlikely miltefosine. Miltefosine is mainly used for visceral leishmaniasis and in some countries to treat CL, but gastrointestinal side effects, teratogenicity alert for young females and hepato- and nephrotoxicity, require patient monitoring; in addition, it was not considered a good candidate for topical treatment [39,40].

## 3. Material and Methods

### 3.1. Botanical Material

The species *Aureliana fasciculata* (Vell.) Sendtner var. *fasciculata* was collected in the city of Simão Pereira, MG, Brazil and was identified by botanist Dr. Rita de Cassia Almeida-Lafetá. A voucher specimen was deposited in RFA Herbarium (UFRJ, Rio de Janeiro, Brazil) under number 40829.

### 3.2. Extraction and Isolation

Leaves were weighed and dried in an oven with circulating air at 40 °C. The dried plant organ was reduced to small fragments in a knife mill (Tecnal 048, Piracicaba, Brazil). After drying, the fragmented leaves were extracted by static maceration at room temperature with methanol. The extract was concentrated by using a rotary evaporator (Buchi, Flawil, Switzerland) into the dry extract. The crude methanol extract (50g) from leaves of *Aureliana fasciculata* var. *fasciculata* was suspended in MeOH/H_2_O (3:7) and subjected to the liquid-liquid partitions in sequences with solvents such as hexane, dichloromethane, ethyl acetate, and butanol. The dichloromethane fraction (2.0 g) was chromatographed on a column of XAD using methanol as eluent. The fractions 2–3 were submitted to chromatography on a column of silica gel, using as solvent systems, binary mixtures of dichloromethane, and methanol in increasing polarity gradient, as also pure hexane, dichloromethane, and methanol. The fractions collected 8–11 were reunited and chromatographed on a preparative plate to obtain the two withanolides: Aurelianolide A (3 mg) and Aurelianolide B (11 mg).

### 3.3. ESI-MS Analysis

Mass spectra were obtained from the High-Resolution device in MicroTOFII Bruker electrospray ionization (Bruker, Bremen, Germany). The samples were diluted with spectroscopic grade MeOH (Tedia, Fairfield, OH, USA) concentration of 500 µg/ml being injected in the device flow 5 min/l. The analysis was performed in the positive mode. Change in spectral window of m/z 50 to 2000. The obtained data were compared to literature data [11].

Aurelianolide A: white amorphous solid. ESI-MS (positive): m/z 551.2625 [M+Na].

Aurelianolide B: white crystals. ESI-MS (positive): m/z 535.2682 [M+Na].

### 3.4. NMR Analysis

Nuclear magnetic resonance spectra of hydrogen and carbon (^13^C and ^1^H NMR) were obtained on Varian device VNMRS-Gemini 500 spectrometer (NMR Associates, Fitchburg, MA, USA) operating at a frequency of 400MHz/100MHz using CD_3_OD as the solvent. Special techniques and bi-dimensional such as COSY, HMBC, and HSQC were also performed. The chemical shift values (δ) in dimensionless units, were referred to an internal standard (TMS), is represented in parts per million (ppm) of the applied frequency for each experiment and coupling constants (J) were measured in Hz. The obtained data were compared to literature data [11].

Aurelianolide A: RMN ^1^H (400MHz, CD_3_OD): (δ, ppm): 6,17 (H2, d, J = 9,92 Hz, 1H), 7,06 (H3, dd, J = 9,98 Hz and 6,28 Hz, 1H), 3,64 (H4, d, J = 6,28 Hz, 1H), 3,15 (H6, s, 1H), 2,07 (H7a, m, 1H), 1,35 (H7b, m, 1H), 1,50 (H8, m, 1H), 1,21 (H9, m, 1H), 1,92 (H11a, m, 1H), 1,47 (H11b, m, 1H), 1,71 (H12a, m, 1H), 1,68 (H12b, m, 1H), 1,75 (H14, m, 1H), 1,89 (H15a, m, 1H), 1,48 (H15b, m, 1H), 5,13 (H16, dd = 8,56 Hz and 2,24 Hz, 1H), 0,85 (H18, s, 3H), 1,37 (H19, s, 3H), 2,25 (H20, dq, J = 6,72 Hz and 4,00 Hz, 3H), 1,04 (H21, d, J = 6,96 Hz, 1H), 4,36 (H22, dt, J = 11,08 and 4,0 Hz, 1H), 2,50 (H23a, m, 1H), 2,12 (H23b, m, 1H), 1,82 (H27, s, 3H), 1,97 (H28, s, 3H), 1,99 (OCH_3,_ s, 3H). RMN ^13^C (100MHz, CD_3_OD): (δ, ppm): 204,17 (C1), 133,17 (C2), 145,32 (C3), 71,14 (C4), 64,75 (C5), 61,16 (C6), 32,46 (C7), 31,08 (C8), 43,60 (C9), 51,73 (C10), 21,78 (C11), 33,24 (C12), 49,67 (C13), 49,25 (C14), 33,78 (C15), 80,13 (C16), 84,46 (C17), 15,34 (C18), 16,94 (C19), 45,22 (C20), 9,59 (C21), 79,94 (C22), 34,59 (C23), 153,19 (C24), 122,15 (C25), 169,20 (C26), 12,39 (C27), 20,50 (C28), 171,61 (COAc), 21,11 (OCH_3_).

Aurelianolide B: RMN ^1^H (400MHz, CD_3_OD): (δ,ppm): 5,86 (H2, d, J = 10,48 Hz, 1H), 6,85 (H3, dd, J = 10,00 Hz and 4,60 Hz, 1H), 4,55 (H4, d, J = 4,60 Hz, 1H), 5,89 (H6, d, J = 4,2 Hz, 1H), 2,07 (H7a, m, 1H), 1,46 (H7b, m, 1H), 1,66 (H8, m, 1H), 1,16 (H9, m, 1H), 2,15 (H11a, m, 1H), 1,55 (H11b, m, 1H), 1,93 (H12a, m, 1H), 1,69 (H12b, m, 1H), 1,86 (H14, m, 1H), 1,90 (H15a, m, 1H), 1,50 (H15b, m, 1H), 5,15 (H16, dd, J = 9,0 Hz and 2,56 Hz, 1H), 0,93 (H18, s, 3H), 1,43 (H19, s, 3H), 2,27 (H20, dq, J = 6,96 Hz and 3,96 Hz, 1H), 1,08 (H21, d, J = 6,96 Hz, 1H), 4,36 (H22, dt, J = 13,92 Hz and 3,96 Hz, 1H), 2,51 (H23a. m, 1H), 2,12 (H23b, m, 1H), 1,83 (H27, s, 3H), 1,97 (H28, s, 3H), 1,99 (OCH_3_, s, 3H). RMN ^13^C (100MHz, CD_3_OD): (δ, ppm): 205,84 (C1), 131,19 (C2), 146,18 (C3), 69,86 (C4), 139,72 (C5), 129,28 (C6), 32,11 (C7), 33,83 (C8), 43,65 (C9), 49,74 (C10), 23,68 (C11), 33,59 (C12), 50,48 (C13), 49,72 (C14), 33,78 (C15), 80,22 (C16), 84,61 (C17), 15,69 (C18), 23,01 (C19), 44,20 (C20), 9,61 (C21), 80,04 (C22), 34,59 (C23), 153,25 (C24), 122,12 (C25), 169,22 (C26), 12,40 (C27), 20,52 (C28), 171,70 (COAc), 21,15 (OCH_3_).

### 3.5. Parasites

*Leishmania amazonensis* promastigotes (MHOM/BR/77/LTB/0016) were maintained at 26°C in Schneider’s medium (Sigma-Aldrich, St. Louis, MO, USA) supplemented with 10% bovine serum (FBS), 100 mg/ml streptomycin and 100 U/ml penicillin. Subcultures were performed twice a week until the seventh passage. Subsequently, old cultures were discarded and fresh parasites were obtained from BALB/c mice lesions.

#### 3.5.1. Antipromastigote Activity

To evaluate the antileishmanial activity, promastigotes of *L. amazonensis* were maintained in cell culture flasks at 26 °C in Schneider’s medium (Sigma-Aldrich, St. Louis, MO, USA), supplemented as described above. Experiments were performed in 96-well plates for 72 h at 26 °C with an initial inoculum of 1.0 × 10^6^ cells/ml and varying concentrations of plant extracts or withanolides. Pentamidine was used as a control, varying from 0.39 to 25µM. After 72h of incubation, parasites viability was assessed adding resazurin (50 µM) for additional 3h. After this time, fluorescence was quantified (excitation λ = 560 nm; emission λ  =  590 nm) and the data obtained from three experiments were expressed as the mean ± standard error of the mean (Mean ± S.E.M.). The half maximal inhibitory concentration (IC_50_) was determined by logarithmic non-linear regression analysis using GraphPrism software (Version 5, GraphPad, San Diego, CA, USA).

#### 3.5.2. Antiamastigote Activity

Resident macrophages were harvested from the peritoneum of BALB/c mice in ice-cold RPMI supplemented with 1% glutamine and 1% pyruvate. The cells were plated at 2.0 × 10^6^/ml (0.4 ml/well) on circular 13mm glass diameter coverslips in 24 well plates and kept in a 5% CO_2_ atmosphere at 37°C, for 1h. Nonadherent cells were removed by washing with pre-warmed complete medium. Macrophages were infected with promastigotes of *L. amazonensis* on stationary phase at a 3:1 parasite/macrophage ratio. After 3h of incubation, the monolayers were washed three times with pre-warmed complete medium to remove free parasites. The withanolides were added in duplicates in concentrations based on the antipromastigote IC_50_, ranging from twice, half and a quarter. The plates were incubated for a further 72 h. Afterward, the coverslips were stained with a Romanowsky stain (Panótico, New Prov, Pinhais, Brazil), according to fabricant instructions. The number of intracellular amastigotes was determined by counting at least 100 macrophages per well. The results were expressed as an infection index (% infected macrophage × number of amastigotes / total number of macrophages) and IC_50_ was determined by logarithmic non-linear regression analysis using GraphPrism software.

### 3.6. Cytotoxic Study

Mouse macrophages cell line J774 were plated at 2.0 × 10^6^ cells/ml in 96-well plates, in ice-cold RPMI supplemented with 10% FBS, 1% glutamine and 1% pyruvate. The cells were incubated at 37°C under an atmosphere of 5% CO_2_ for 1 h. Non-adherent cells were removed by washing with pre-warmed complete medium. Tests were performed with concentration ranging from 0 to 100 µM of withanolides. After 72h of incubation, macrophages viability was measured by colorimetric assay using rezasurin (50µM). After 3 h, the fluorescence was quantified (excitation λ = 560 nm; emission λ  =  590 nm) and CC_50_ value (concentration that reduces in 50% the cells viability) was determined by logarithmic non-linear regression analysis using GraphPrism software. Selectivity index (SI) was expressed by the ratio between CC_50_ value over the host cells and the IC_50_ obtained over intracellular amastigotes.

### 3.7. Nitric Oxide Production

Supernatant from antiamastigote assay was collected after 72h and the nitric oxide (NO) concentration was indirectly measured using Griess reagent, as described by Green et al. [41]. Griess reagent is 0.1% *N*-(1-Naphthyl) ethylenediamine (under orthophosphoric acid conditions−5%) and 1% sulfanamide solution. The reaction was realized by the addition of 50µl of Griess reagent and 50µl of supernatants obtained from the antiamastigote assay. After 10 min of incubation at room temperature, the absorbance at 540 nm was measured and the nitrite concentration was determined from a sodium nitrite (NaNO_2_) solution standard curve.

### 3.8. In Silico ADMET properties

We performed some theoretical analysis of the drug-likeness of withanolides. The pharmacokinetic profile of a compound defines its absorption, distribution, metabolism, excretion, and toxicity (ADMET). The ADMET properties of withanolides were evaluated using the admetSAR tool [42] and the Lipinski’s rule of the compounds was also calculated.

## 4. Conclusions

*Aureliana fasciculata* (Vell.) Sendtner var. *fasciculata* is a Brazilian species of Atlantic Forest that has been no longer investigated on the chemical and biological activity point of view. The phytochemical study of *A. fasciculata* var. *fasciculata* (Vell.) Sendtner var. *fasciculata* resulted in the isolation of two potential leishmanicidal withanolides: Aurelianolide **A** and Aurelianolide **B**, which show the trend of the species subfamily Solanoideae to withanolides in the family Solanaceae.

When outlining a strategy to screen the antileishmanial activity of plant extracts or other compounds, the first step involves choosing an approach: target-driven or phenotypic assays. Both assays have advantages and disadvantages. The target-driven assay allows to detect compounds with a mechanism of action previously chosen but limited to only one target. Phenotypic assay allows to explore all the molecular targets in whole and live parasites. However, when an active compound is found, the discovery of the mechanism of action is challenging. Here we decided to use a phenotypic assay with promastigotes and intracellular amastigotes to maximize the probability of finding an active compound. The extraction guided by the antileishmanial activity revealed Aureanolides A and B as the active compounds present in *A. fasciculata*.

These compounds showed direct activity on the parasite, as shown by antipromastigote assay, but the lower IC_50_ for intracellular amastigotes and the enhancement in NO production in the highest concentration suggest also an additional mechanism involving the host cell. The in-silico predictions pointed to a high probability for good bioavailability by oral route and low toxicity. The scaffold of aurelianolides and the findings of its in-silico pharmacokinetics properties are the major guides for optimization possibilities that could support their future preclinical and clinical applications to leishmaniasis. The low VDss might suggest plasma protein binding (PPB) and this phenomenon influences the absorption, biodistribution, metabolism, and excretion of drugs. In silico results of our study also corroborate with the one performed by Singh et al. [43] in which withanolide A from *Withania somnifera* (L.), with neuropharmacological activity, presented high PPB, passive permeability and a fast and wide distribution kinetics in vitro. Also, Dubay et al. [44] demonstrated that withanolides and withanosides of *W. somnifera* have a strong binding to serum albumin. In addition, further structure-based drug design with withanolides is required. Initially, to improve the distribution volume and make aurelianolides more available to the tissues affected by the high parasitic load in patients with leishmaniasis, changes in the structure to increase liposolubility, without resulting in the loss of its biological effect, should be performed. Along with that, combined techniques to pursue their molecular target like scaffold-based virtual ligand screening and Quantitative structure–activity relationship (QSAR) are also complementary attempts that could help us to find a ligand and exploit pharmacokinetic and pharmacodynamics properties of aurelianolides to achieve an improved lead compound.

## Figures and Tables

**Table 1:** Antileishmanial activity, cytotoxicity, and selectivity index for fractions and withanolides from *Aureliana fasciculata*.

	*L. amazonensis*		J774 Macrophages (CC_50_) *	Selectivity Index (SI)
	Promastigotes (IC_50_) *	Intracellular Amastigotes (IC_50_) *		
AFfPH	26.6 ± 0.1	N.D.	N.D.	N.D.
AFfDi	1.9 ± 0.7	N.D.	N.D.	N.D.
AFfAc	11.4 ± 0.1	N.D.	N.D.	N.D.
AFfBu	13.0 ± 0.1	N.D.	N.D.	N.D.
Aurelianolide A	4.0 ± 0.1 (7.6 ± 0.1)	1.2 ± 0.1 (2.3 ± 0.1)	6.7 ± 0.2 (12.7 ± 0.2)	5.6
Aurelianolide B	4.1 ± 0.3 (7.9 ± 0.7)	3.3 ± 0.1 (6.4 ± 0.1)	6.7 ± 0.1 (13.1 ± 0.1)	2.0
Pentamidine	2.8 ± 0.1 (4.8 ± 0.1)	1.1 ± 0.1 (1.9 ± 0.1)	5.0 ± 0.7 (8.5 ± 1.2)	4.5

SI = CC_50_/IC_50_ in amastigotes; * µg/ml (µM).

**Table 2:** Physicochemical parameters and Lipinski’s rule of five of withanolides and miltefosine using pkCMS tool*.

Parameters	Aurelianolide A	Aurelianolide B	Miltefosine
MW	528.642	512.643	407.576
LogP	3.037	3.825	5.6755
#ACCEPTORS	8	7	4
#DONORS	2	2	0
Water solubility (log mg/l)	−4.924	−5.329	−5.673

* MW, molecular weight; LogP, logarithm of the compound partition coefficient between *n*-octanol and water; #, NHB (number of hydrogen bonds)

**Table 3:** In silico ADME (absorption, distribution, metabolism, excretion) properties of Aurelianolides and miltefosine using pkCMS tool *.

Parameters	Aurelianolide A	Aurelianolide B	Miltefosine
*Absorption*			
Caco2 permeability (log Papp in 10^−6^ cm/s)	1.31	1.474	1.153
Intestinal absorption (human, %)	91.459	90.543	94.987
Skin Permeability (log Kp)	−3.101	−3.638	−2.702
*Distribution*			
VDss (human, l/kg)	0.04	0.121	0.96
Fraction unbound (human)	0.223	0.171	0.238
BBB permeability (log BB)	−0.801	−0.602	−0.345
CNS permeability (log PS)	−3.278	−3.033	−3.172
*Metabolism*			
CYP2D6 substrate	No	No	No
CYP3A4 substrate	Yes	Yes	Yes
CYP1A2 inhibitor	No	No	No
CYP2C19 inhibitor	No	No	No
CYP2C9 inhibitior	No	No	No
CYP2D6 inhibitior	No	No	No
CYP3A4 inhibitior	No	No	No
*Excretion*			
Total Clearance (log ml/min/kg)	0.275	0.36	1.156

* VDss, steady-state volume of distribution; BBB, blood-brain barrier; CNS, central nervous system.

**Table 4:** In silico Toxicity of Aurelianolides and miltefosine using the pkCMS tool.

Parameters	Aurelianolide A	Aurelianolide B	Miltefosine
AMES toxicity	No	No	No
Max. tolerated dose (human, log mg/kg/day)	−1.053	−0.858	1.079
hERG I inhibitor	No	No	No
hERG II inhibitor	Yes	Yes	Yes
Oral Rat Acute Toxicity (LD50) (mol/kg)	2.518	2.284	2.211
Oral Rat Chronic Toxicity (LOAEL) (log mg/kg_bw/day)	1.786	1.692	1.34
Hepatotoxicity	No	No	Yes
Skin Sensitisation	No	No	No
*T. Pyriformis* toxicity pIGC50 (log µg/l)	0.299	0.340	1.054
Minnow toxicity LC50 (log mM)	0.503	0.109	−2.403

## References

[1] Alvar J., Vélez I.D., Bern C., Herrero M., Desjeux P., Cano J., Jannin J., den Boer M., WHO Leishmaniasis Control Team (2012). Leishmaniasis worldwide and global estimates of its incidence. PLoS ONE.

[2] Sundar S., Singh A. (2017). Chemotherapeutics of visceral leishmaniasis: Present and future developments. Parasitology.

[3] de Vries H.J.C., Reedjik S.H., Schallig H.D.F.H. (2015). Cutaneous Leishmaniasis: Recent developments in diagnosis and management. Am. J. Clin. Dermatol..

[4] Berman J.N. (1998). Chemotherapy of leishmaniasis: Recent advances in the treatment of visceral disease. Curr. Opin. Infect Dis..

[5] Gontijo B., Carvalho M.L. (2003). American cutaneous leishmaniasis. Ver. Soc. Bras. Med. Trop..

[6] Jhingran A., Chawla B., Saxena S., Barret M.P., Madhubala R. (2009). Paronomycin: Uptake and resistance in *Leishmania donovani*. Mol. Biochem. Parasitol..

[7] Souza V.C., Lorenzi H. (2005). Botânica Sistemática: Guia ilustrado para identificação das famílias de Angiospermas da flora brasileira, baseado em APG II..

[8] Elabbar F.A., Nawill M.A.B., Ashraf T.M.E. (2014). Extraction, separation and identification of compounds from leaves of *Solanum elaeagnifolium* Cav. (Solanaceae). Int. Curr. Pharm. J..

[9] Hawkes J.G. (1999). The economic importance of the family Solanaceae, In Solanaceae IV. Advances in Botany and Utilization.

[10] Hunziker A.T., Barbosa G. (1991). Estudios sobre Solanaceae XXX: Revisión de Aureliana. Darwiniana.

[11] Almeida-Lafetá R., Ferreira M.J.P., Emerenciano V.P., Kaplan M.A.C. (2010). Withanolides from *Aureliana fasciculata var. fasciculata*. Helv. Chim. Acta.

[12] Dhar N., Razdan S., Rana S., Bhat W.W., Vishwakarma R., Lattoo S.K.A. (2015). Decade of molecular understanding of withanolide biosynthesis and in vitro studies in *Withania somnifera* (L.) Dunal: Prospects and perspectives for pathway engineering. Front. Plant Sci..

[13] Vaishnavi K., Saxena N., Shah N., Singh R., Manjunath K., Uthayakumar M., Kanaujia S.P., Kaul S.C., Sekar K., Wadhwa R. (2012). Differential activities of the two closely related withanolides, withaferin A and withanone: Bioinformatics and experimental evidences. PLoS ONE.

[14] Olmstead R.G., Bohs L., Migid H.A., Santiago-Valentin E., Garcia V.F., Collier S.M. (2008). A molecular phylogeny of the Solanaceae. Taxon.

[15] Zhang H., Samadi A.K., Cohen M.S., Timmermann B.N. (2012). Antiproliferative withanolides from the Solanaceae: A structure–activity study. Pure Appl. Chem..

[16] Glotter E. (1991). Withanolides and related ergostane-type steroids. Nat. Prod. Rep..

[17] Kim K.H., Choi S.U., Choi S.Z., Son M.W., Lee K.R. (2011). Withanolides from the rhizomes of *Dioscorea japonica* and their cytotoxicity. J. Agric. Food Chem..

[18] Chao C.H., Chou K.J., Wen Z.H., Wang G.H., Wu Y.C., Dai C.F., Sheu J.H. (2011). Paraminabeolides A.−F, cytotoxic and anti-inflammatory marine withanolides from the soft coral *Paraminabea acronocephala*. J. Nat. Prod..

[19] Kaileh M., Vanden B.W., Heyerick A., Horion J., Piette J., Libert C., De Keukeleire D., Essawi T., Haegeman G. (2007). Withaferin A strongly elicits I kappa B kinase beta hyperphosphorylation concomitant with potent inhibition of its kinase activity. J. Biol. Chem..

[20] Mulabagal V., Subbaraju G.V., Rao C.V., Sivaramakrishna C., DeWitt D.L., Holmes D., Sung B., Aggarwal B.B., Tsay H.S., Nair M.G. (2009). Withanolide sulfoxide from aswagandha roots inhibits nuclear transcription factor-kappa-B, cyclooxygenase and tumor cell proliferation. Phytother. Res..

[21] Nagafuji S., Okabe H., Akahane H., Abe F. (2004). Trypanocidal constituents in plants. Bio Pharm. Bull..

[22] Chandrasekaran S., Dayakar A., Veronica J., Sundar S., Maurya R. (2013). An in vitro study of apoptotic like death in *Leishmania donovani* promastigotes by withanolides. Parasitol. Int..

[23] Budhiraja R.D., Krishan P., Sudhir S. (2000). Biological activity of withanolides. J. Sci. Ind. Res..

[24] Nicolás F.G., Reyes G., Audisio M.C., Uriburu M.L., González S., Barboza G.E., Nicotra V.E. (2015). Withanolides with antibacterial activity from Nicandra john-tyleriana. J. Nat. Prod..

[25] Misico R.I., Viviana E., Nicotra J.C., Oberti G.B., Gil R.R., Burton G. (2011). Withanolides and related steroids. Prog. Chem. Org. Nat. Prod..

[26] Kuroyanagi M.I., Murata M., Nakane T., Shirota O., Sekita S., Fuchino H., Shinwari Z.K. (2012). Leishmanicidal activity withanolides from a Pakistani medicinal plant, *Withania coagulans*. Chem. Pharm. Bull..

[27] Choudary M.I., Yousaf S., Ahmed S., Samreen Y.K., Rahman A. (2005). Antileishmanial physalins from *Physalis minima*. Chem. Biodivers..

[28] Bravo B.J.A., Sauvain M., Gimenez T.A., Balanza E., Serani L., Laprévote O. (2001). Trypanocidal withanolides and withanolide Glycosides from *Dunalia brachyacantha*. J. Nat. Prod..

[29] Murray H.W., Nathan C.F. (1999). Macrophage microbicidal mechanisms in vivo: Reactive nitrogen versus oxygen intermediates in the killing of intracellular visceral *Leishmania donovani*. J. Exp. Med..

[30] Bogdan C., Rollinghoff M., Diefenbach A. (2000). Reactive oxygen and reactive nitrogen intermediates in innate and specific immunity. Curr. Opin. Immunol..

[31] Carneiro P.P., Conceição J., Macedo M., Magalhães V., Carvalho E.M., Bacellar O. (2016). The role of nitric oxide and reactive oxygen species in the killing of *Leishmania braziliensis* by monocytes from patients with Cutaneous Leishmaniasis. PLoS ONE.

[32] Yang B.Y., Guo R., Li T., Liu Y., Wang C.F., Shu Z.P., Wang Z.B., Zhang J., Xia Y.G. (2014). Five withanolides from the leaves of *Datura metel* L. and their inhibitory effects on Nitric Oxide production. Molecules.

[33] Lipinski C.A., Lombardo F., Dominy B.W., Feeney P.J. (1997). Experimental and computational approaches to estimate solubility and permeability in drug discovery and development settings. Adv. Drug Deliv. Rev..

[34] Sundar S., Olliaro P.L. (2007). Miltefosine in the treatment of leishmaniasis: Clinical evidence for informed clinical risk management. Ther. Clin. Risk Manag..

[35] McKerrow J.H., Lipinski C.A. (2017). The rule of five should not impede anti-parasitic drug development. Int. J. Parasitol. Drugs Drug Resist..

[36] Bhattacharya S.K., Sinha P.K., Sundar S., Thakur C.P., Jha T.K., Pandey K., Das V.R. (2007). Phase 4 trial of miltefosine for the treatment of Indian visceral leishmaniasis. J. Infect. Dis..

[37] Field M.C., Horn D., Fairlamb A.H., Ferguson M.A., Gray D.W., Read K.D., De Rycker M., Torrie L.S., Wyatt P.G., Wyllie S. (2017). Anti-trypanossomtid drug discovery: An ongoing challenge and a continuing need. Nat. Rev. Microbiol..

[38] Soto J., Rea J., Balderrama M., Toledo J., Soto P., Valda L., Berman J.D. (2008). Efficacy of miltefonsine for Bolivian cutaneous leishmaniasis. Am. J. Trop. Med. Hyg..

[39] Uranw S., Ostyn B., Dorlo T.P., Hasker E., Dujardin B., Dujardin J.C., Rijal S., Boelaert M. (2013). Adherence to miltefosine treatment for visceral leishmaniasis under routine conditions in Nepal. Trop. Med. Int. Health.

[40] Van Bocxlaer K., Yardley V., Murdan S., Croft S.L. (2016). Opical formulations of miltefosine for cutaneous leishmaniasis in a BALB/c mouse model. J. Pharm. Pharmacol..

[41] Green L.C., Wagner D.A., Glogowski J., Skipper P.L., Wishnok J.S., Tannebaum S.R. (1982). Analysis of nitrate, nitrite, and [15N] nitrate in biological fluids. Anal. Biochem..

[42] Cheng F., Li W., Zhou Y., Shen J., Wu Z., Liu G., Lee PW. (2012). AdmetSAR: A comprehensive source and free tool for assessment of chemical ADMET properties. J. Chem. Inf. Model..

[43] Singh S.K., Valicherla G.R., Joshi P., Shahi S., Syed A.A., Gupta A.P., Hossain Z., Italiya K., Makadia V., Singh S.K., Wahajuddin M., Gayen J.R. (2018). Determination of permeability, plasma protein binding, blood partitioning, pharmacokinetics and tissue distribution of Withanolide A in rats: A neuroprotective steroidal lactone. Drug Dev Res..

[44] Dubey S., Kallubai M., Sarkar A., Subramanyam R. (2018). Elucidating the active interaction mechanism of phytochemicals withanolide and withanoside derivatives with human serum albumin. PLoS ONE.

